# A systematic review of transphyseal ACL reconstruction in children and adolescents: comparing the transtibial and independent femoral tunnel drilling techniques

**DOI:** 10.1186/s40634-023-00577-0

**Published:** 2023-01-25

**Authors:** Wolf Petersen, Sebastian Bierke, Amelie Stöhr, Thomas Stoffels, Martin Häner

**Affiliations:** 1 Klinik für Orthopädie und Unfallchirurgie, Martin Luther Krankenhaus, Caspar Theyß Strasse 27-31, 14193 Berlin, Grunewald, Berlin Germany; 2 Orthopädische Chirurgie München, Munich, Germany; 3 OC Stadtmitte, Berlin, Germany

**Keywords:** ACL injury, ACL repair, Medial portal drilling, Anatomical ACL reconstruction, Knee

## Abstract

**Purpose:**

Aim of this systematic review was to analyze the outcome after transphyseal ACL reconstruction in children and adolescents regarding the femoral drilling technique.

**Methods:**

A systematic literature search was carried out in various databases on studies on transphyseal ACL reconstruction in children and adolescents. The literature search was limited to the last 20 years. Primary outcome criterion was the failure rate. Secondary outcome criteria were growth disturbances such as leg length discrepancies or deformities and clinical scores.

The present study was registered prospectively (www.crd.york.ac.uk/PROSPERO; CRD42022345964).

**Results:**

A total of 22 retrospective or prospective case series (level 4 evidence) were identified that reported on transphyseal ACL reconstruction in children and adolescents. The overall failure/rupture rate after transphyseal ACL reconstruction was 11.0%. The overall ACL rupture rate of the contralateral side was 9.7%. No statistical significant difference in the failure rates between independent and transtibial drilling techniques could be detected ((*p* = 0.76/*p* = 0.28)). Furthermore no statistical significant differences in the rate of reported growth disturbances between independent and transtibial drilling techniques were shown (*p* = 0.15). The reported clinical scores at follow-up (mean follow-up 5.05 years) revealed good to very good results.

**Conclusion:**

This systematic review demonstrates that children and adolescents have a relatively high failure rate after transpyseal ACL reconstruction without any statistically significant differences between independent or transtibial drilling techniques regarding reruptur rates or the rate of growth disturbances. The results of this systematic review warrant a comparison of both techniques for femoral tunnel drilling in a controlled randomized trial.

## Level of evidence: IV

### Introduction

Anterior cruciate ligament (ACL) injury is a common knee injury in physically active children and adolescents. According to a recent study from Finland, the incidence is 23.3 per 100,000 person-years [1].

A problem of childhood ACL injury is the high rate of secondary meniscus and cartilage damage. In the long term, these can lead to the development of premature osteoarthritis (OA) [2–4].

However, treatment of pediatric ACL injuries is controversial mainly due to the potential risk of growth disorders in surgical treatment [5].

Due to the high risk of knee OA, the indication for reconstruction of the anterior cruciate ligament is seen in children with accompanying meniscal lesions, in recurring giving way episodes or in the case of an unacceptable restriction in sporting activities [6].

Various techniques have been described for ACL reconstruction in children. A widely used surgical technique is the transphyseal ACL reconstruction, since a previous systematic review has shown the risk of growth plate disturbance to be higher with physeal-sparing techniques [7].

In the past, when using transphyseal techniques, care was taken to ensure that the femoral drill tunnel was as steep as possible in order to protect Ranvier's zone [6, 8]. An experimental study in sheep has shown that injury to the Ranvier zone is associated with disturbances in the growing skeleton [8].

Steep femoral tunnels are usually created by transtibial drilling. Since these bone tunnels are often outside the femoral insertion zone of the original ACL, they are also referred to as non-anatomical [9]. A problem with the non-anatomical tunnel position is that rotational stability is not restored as well as with an anatomical tunnel position [10, 11]. The prevention of osteoarthritis is said to be better with anatomical than with non-anatomical surgical techniques [12]. Therefore, an anatomical technique is recommended for ACL reconstruction in adults [13].

One important criterion of an anatomical ACL reconstruction is drilling the femoral bone tunnel independent from the tibial tunnel (medial portal or outside in technique) because the risk of a non-anatomical tunnel placement is higher when using the transtibial technique. A recent meta-analysis of 22 randomized controlled trials about ACL reconstruction in adults has shown that both the International Knee Documentation Committee (IKDC) subjective score and Lysholm score were higher with an independent drilling approach compared to the transtibial drilling technique [14]. However, in this meta-analysis no difference was documented in terms of the risk of reinjury. A comparative study on ACL reconstruction in children with open growth plates cannot be found in literature.

Therefore, it is the aim of this systematic review to compare studies on transphyseal ACL reconstruction with transtibial or independent drilling techniques regarding re-rupture rate (primary outcome criterium), growth disorders and clinical scores (secondary outcome criteria).

Our hypothesis is that no difference in re-rupture rates and clinical scores between transtibial and independent drilling techniques when creating the femoral tunnel in ACL reconstruction in children can be found. However, we hypothesize that the rate of growth abnormalities such as leg length discrepancies and angular deformities is higher in studies on independent drilling techniques.

## Methods

### Search details

Between August 15, 2022 and September 30, 2022, a systematic literature search was carried out in various databases (PubMed, MEDLINE, EMBASE, Scopus, Google scholar) according to PRISMA criteria in order to identify studies examining the outcome after transphyseal reconstruction of the anterior cruciate ligament (ACL) in children and adolescents. The main search was carried out by two reviewers (MH and WP).

The following search terms were used: ACL reconstruction in children, ACL reconstruction and skeletally immature patients, ACL reconstruction and open physis, ACL reconstruction and open growth plates, ACL reconstruction in children with open physis, ACL reconstruction and skeletally immature, and pediatric ACL reconstruction.

If a corresponding study was found, related articles were researched in the various databases and relevant publications were searched for. In addition, the reference section of relevant studies was also checked for relevant citations.

Inclusion and exclusion criteria were defined for article selection. Inclusion criteria were: 1. transphyseal ACL reconstruction in children, and 2. English language. Exclusion criteria were: 1. inclusion of patients older than 16 years, 2. drilling technique not clearly stated, 3. not clearly described how many patients were treated with the different drilling techniques, 4. no failure rate reported, 5. less than 10 participants, 6. previous systematic reviews, previous meta-analysis, 7. follow-up < 2 years, 8. extraarticular reconstruction technique and 9. publication date < 2002.

If one patient group in a study met the inclusion criteria and another patient group did not, then only the group that met the inclusion criteria was included in the current analysis. When multiple articles of one clinical trial were available, the trial with the longest follow-up was included.

The present study was registered prospectively (www.crd.york.ac.uk/PROSPERO; CRD42022345964).

### Risk of bias (quality) assessment

The quality assessment tool should be used depending on the study type. The PEDRO scale was used in case of randomized controlled trials and the Methodological Index for Non-Randomized Studies (MINORS) for non-randomized clinical trials [15].

Quality assessment was conducted by W.P. and M.H.

### Data extraction (selection and coding)

After researching the literature according to the specified inclusion and exclusion criteria, W.P. and M.H. extracted the following data from the selected studies:

1. study details: journal of publication, date of publication, sample size, study design, follow-up, inclusion and exclusion criteria
2. patient details: age, gender
3. surgical details: drilling technique, graft source
4. outcome measures: failure rate, rate of growth disturbances, patient reported outcome measures (PROMs).

### Outcome measures

Primary outcome measure was the failure rate/rupture rate of ACL reconstruction as reported by the authors. Secondary outcome measures were, the rate of contralateral ACL ruptures, the rate of growth disturbances such as leg length discrepancies or varus and valgus deformities and various patient reported outcome measures (PROMs) such as Lysholm score, IKDC Score, or Tegner activity score. Growth disturbances were classified into those requiring further treatment and those requiring no further therapy. All growth disturbances reported in each study were summarized in tables.

### Strategy for data synthesis

W.P. and M.H. have constructed a narrative synthesis of the extracted data, structured around the failure rate, rate of growth disturbances, and PROMS. Tables have been developed to aid the presentation of the extracted data along with the quality assessment. The primary goal of this systematic review was to compare transtibial and independent femoral drilling techniques. A secondary aim of this study was to compare different graft sources such as hamstring tendon grafts (HT), bone patellar bone grafts (BTB) and quadriceps tendon grafts (QT) regarding re-rupture rate. A formal meta-analysis was performed for the primary outcome measure (failure rate), for the rate of contralateral ACL ruptures and for growth disturbances which required treatment. For the cumulative failure rates number of patients and number of failures for both different groups (transtibial drilling and independent drilling, graft types) were summarized and the overall numbers and percentages for both procedures were calculated.

### Statistics

The overall failure rate was calculated using the total number of subjects in the included studies and the number of reported failures. The students t-test was applied for the comparison of calculated failure rates of different inside-out implants. Calculation of the 95% confidence interval was used to compare calculated failure rates of different grafts used for ACL reconstruction.

## Results

### Search results and study design

**Fig. 1:**
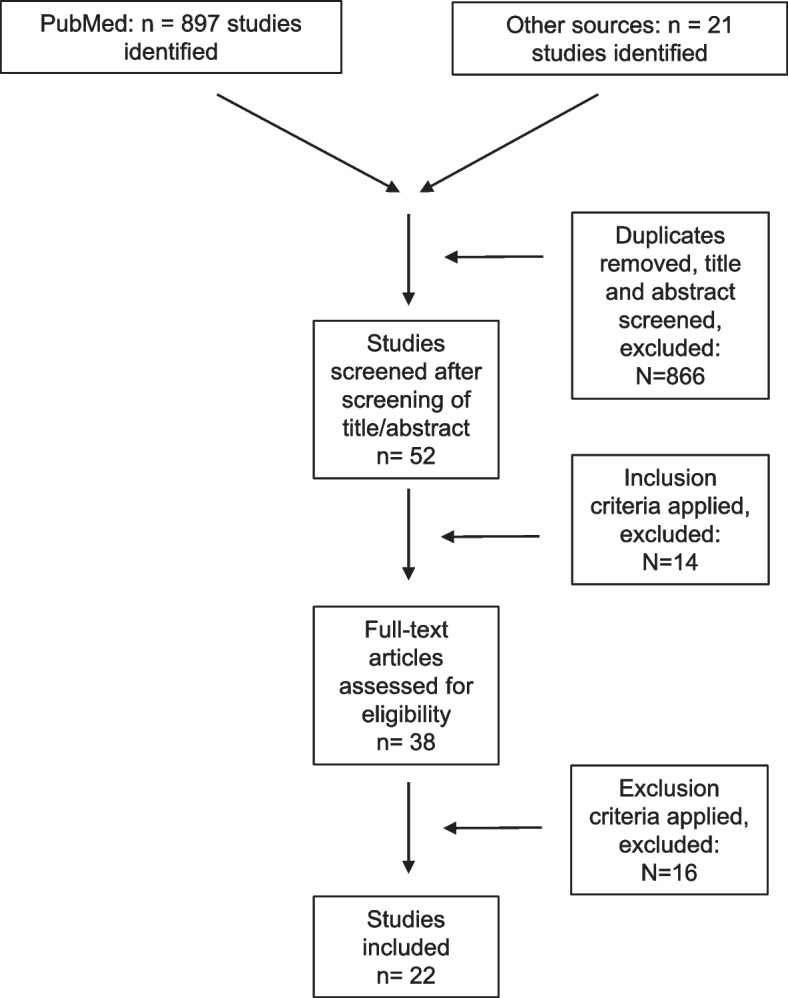
Flow chart of literature search and review process

The search results are shown in Fig. 1. Out of 38 studies that met the inclusion criteria, 16 studies had to be excluded due to the exclusion criteria. All included studies were retrospective or prospective cohort studies. No randomized controlled clinical trial about ACL reconstruction in children could be found. Detailed information about study designs and quality is provided in Table 1.

**Table 1:** Studies on transphyseal ACL reconstruction

**Nr**	**First author, year**	**No. of patients**	**Age of patients** **(years)**	**Study design**	**MINORS**	**Femoral drilling technique**	**Graft**	**Tanner stage**	**Mean follow up (years)**	**Re-rupture (%)**	**Contralateral ACL rupture (%)**	**Growth abnormalities with need for treatment**
1	Shelbourne, 2004	16	14.8	Retrospective	9	TT	BTB	3–4	3.4	1 (6.3%)	2 (12.5%)	0
2	McIntosh, 2006	18	13.8	Retrospective	8	TT	HT	NR	3.4 (2–9.3)	2 (11.1%)	NR	1
3	Liddle, 2008	17	12.1	Retrospective	7	TT	HT	NR	4.4	1 (5.9%)	NR	0
4	Cohen, 2009	26	13.3	Retrospective	7	TT	HT	NR	4.5	3 (11.5%)	NR	0
5	Streich et al. 2010	16	11	Retrospective	9	TT	HT	1–2	5.8	0 (0)	NR	0
6	Mauch, 2011	49	13	Retrospective	10	TT	QT	NR	5	5 (10.2%)	5 (10.2%)	1
7	Nikolaou, 2011	94	13.7	Retrospective	10	TT	HT	NR	3.2 (2–5)	4 (4.3%)	NR	0
8	Courvoisier, 2011	37	14	Retrospective	6	TT	HT	NR	2	5 (13.5%)	NR	0
9	Hui, 2012	16	12	Prospective	13	MP	HT from living donor	NR	2.1 (1.8–2.8)	0 (0)	0 (0)	0
10	Redler, 2012	18	14.2	Retrospective	**9**	TT	HT	NR	3.6 (2–7.2)	0 (0)	3 (16.6%)	0
11	Memeo 2012	10	14.4	Retrospective	7	TT	BTB	3	15	1 (10.0%)	NR	0
12	Kumar, 2013	32	11.3	Prospective	9	TT	HT	1–3	6 (4–10.8)	1 (3.1%)	1 (3.1%)	0
13	Kohl, 2014	15	12.8	Prospective	9	TT	QT	2–4	4 (2–8)	0 (0)	NR	0
14	Calvo et al. 2015	27	13	Retrospective	10	TT	HT	2–4	10.6 (10–13)	5 (18.5%)	NR	0
15	Fauno, 2016	39	11.7	Prospective	11	TT	HT	NR	5.6. ± 2.8	1 (2.6%)	NR	0
16	Siebold, 2016	35	13.6	Retrospective	9	MP	HT	NR	3.4 + -1.3	9 (26.7%)	NR	0
17	Placella,2016	24	13.2 (	Retrospective	9	All inside	HT	1–5	8	0 (0)	NR	0
18	Pennoc, 2019	90	14.8	Retrospective	12	Outside in	QT (27), HT (53)	NR	2.8	13 (14.4%) QT: 1 (3.7%) HT: 12 (22.6%)	10 (11.1%) QT: 5 (18.5%) HT: 5 (9.4%)	0
19	Astur, 2019	52	13.6	Retrospective	8	TT	HT	NR	7.4	18 (34.6%)	NR	0
20	Gosh, 2020	100	14 (8–16)	Prospective	12	MP	HT from living donor	1–5	2	12 (12.0%)	9 (9.0%)	0
21	Hanssen, 2022	193	12.5 (7–15)	Prospective	11	TT	HT	NR	6.9 (5–9)	24 (12.4%)	23 (11.9%)	NR
22	Kilkenny, 2022	86	12–15	Retrospective	10	MP	HT (59) BTB (27)	NR	2	6 (7.0%) HT: 6 (10.2) BTB: 0 (0)	5 (5.8%) HT: 3 (5.1%) BTB: 2 (7.4%)	NR
	**Total**	**1010**								**111 (11.0%)**	**58 (9.7%)**	**2 (0.2%)**

7

*TT* Transtibial, *MP* Medial portal, *BTB* Bone patellar tendon bone, *HT* Hamstring tendon, *QT* Quadriceps tendon, *NR* Not reported

A total of 22 articles were identified that reported failure rates of transphyseal ACL reconstruction in children younger than 16 years [16–37]. In 16 studies (659 patients) a transtibial technique and in 6 studies (351 patients) an independent technique was used for drilling the femoral tunnel (Table 1).

Autologous hamstring tendons (HT) were utilized in 14 studies, whereas 3 studies each used autologous bone patellar tendon bone (BTB) or autologous quadriceps tendon (QT) grafts (49 with bone plug, 42 without bone plug). HT from living donors were expended in 2 studies. No study reported the use of allografts (Table 1).

### Failure rate/rupture rate

A total of 111 failed ACL reconstructions (11.0%) and 58 contralateral ACL ruptures (9.7%) were described in all included studies (Table 2). The re-ruptur rate ipsilateral varied between 0% and 34.6% and the rupture rates for contralateral ACL varied between 0% and 16.6% (Table 1).

**Table 2:** Comparison of re-rupture rates between transtibial and independent femoral drilling techniques

Femoral drilling technique	Re-rupture ipsilateral	ACL rupture contralateral
Transtibial drilling (A)	**71 (10.8%)**	**34 (10.5%)**
Independent drilling (B)	**40 (11.4%)**	**24 (8.7%)**
Odds/Ratio	**0.94**	**0.74**
95% CI	**0.6223 to 1.4162**	**0.4322 to 1.2711**
Z Statistic	**0.301**	**1.088**
Significance level	***p* = 0.7634**	***p* = 0.2765**

A comparison of the failure rates showed no difference between transtibial and independent femoral drilling techniques (Table 2). Further, no difference between the different drilling techniques was identifiable regarding contralateral ACL ruptures (Table 2).

However, there was a difference in failure rates of the individual grafts (Table 3). The highest failure rate was observed in studies using HT (all using semitendinosus and gracilis tendon), whereas the lowest failure rate could found in the studies using BTB (Table 3). Most of the HT were fixated extracortical. The rate of contralateral ACL ruptures was described to be higher in studies using QT grafts in comparison to the other graft choices (Table 3).

**Table 3:** Comparison of re-rupture rates of different grafts used in the included studies

Graft source	Re rupture ipsilateral	ACL rupture contralateral
HT	**97 (13.1%)**	**35 (9.2%)**
HT from living donor	**12 (10.3%)**	**9 (7.8%)**
BTB	**2 (3.8%)**	**4 (9.3%)**
QT	**6 (6.5%)**	**10 (13.2%)**
Allograft	**NR**	**NR**

*HT* Hamstring tendon, *BTB* Bone patellar bone tendon, *QT* Quadriceps tendon, *NR* Not reported

### Growth abnormalities

Of all 22 studies, 20 studies examined growth disturbances such as leg length discrepancies or deformities in the coronary plane (Tables 4 and 5). In eleven studies long leg standing x rays were used and in 5 studies short radiographs were applied. Two studies described clinical investigation of leg length and valgus/varus deformity and if positive radiographs were performed. Another study combined clinical examination with MRI and one study practiced only clinical examination (Tables 4 and 5).

**Table 4:** Leg length discrepancies in studies using transtibial femoral drilling technique

Nr	First author, year	Examination method	Leg length discrepancy ≥ 1.5 cm	Angular deformity ≥ 5°	Treatment for growth disturbance
1	Shelbourne, 2004	Clinical and short radiographs	0	0	NR
2	McIntosh, 2006	Clinical and if pathological long standing x rays	1	0	Internal shoe lift
3	Liddle,2008	Clinical	0	1	NR
4	Cohen, 2009	Clinical and radiological, long leg x ray	0	0	NR
5	Streich et al. 2010	Clinical and radiological, short x rays	0	0	NR
6	Nikolaou, 2011	Clinical and if positive radiological long leg	0	0	NR
7	Mauch, 2011	Clinical and radiological, long leg x ray	0	1	Distal femoral osteotomy
8	Courvoisier, 2011	Clinical and radiological, long leg x ray	0	0	NR
9	Redler, 2012	Clinical and radiological, short x rays	0	0	NR
10	Memeo, 2012	Clinical and radiological, short x rays	0	0	NR
11	Kumar, 2013	Clinical and radiological, short x rays	0	1	NR
12	Kohl, 2014	Clinical and radiological, long leg x ray	2	1	NR
13	Calvo, 2015	Clinical and radiological, long leg x ray	0	0	NR
14	Fauno, 2016	Clinical and radiological, long leg x ray	NR (9 patients > 1 cm)	0	NR
15	Astur, 2019	Clinical and radiological, long leg x ray	0	0	NR

**Table 5:** Leg length discrepancies in studies using independent femoral drilling technique

Nr	First author, year	Examination method	Leg length discrepancy ≥ 1.5 cm	Angular deformity ≥ 5°	Treatment for growth abnomailty
1.	Hui, 2012	Clinical and radiological, long leg x ray	0	0	NR
2.	Placella, 2016	Clinical and radiological, long leg x ray	0	0	NR
3.	Siebold, 2016	Clinical and radiological, MRI	0	0	NR
4.	Pennock, 2019	Clinical and radiological, long leg x ray	0	0	NR
5.	Gosh, 2020	Clinical and short radiographs	0	0	NR

Table 4 shows that studies using a transtibial drilling technique reported 3 cases with a leg length discrepancy of more than 1.5 cm and 4 cases with an angular valgus deformity of more than 5°. Another study notified leg length discrepancy of more than 1 cm in 9 patients. No study displayed any angular varus deformity. One study reported that a femoral osteotomy was needed to correct the angular deformity and in one study an internal shoe lift was prescribed. In studies using the independent drilling technique no leg length discrepancy or angular deformity were described (Table 5). There was no statistically significant difference between the two groups (*p* = 0.15).

### Stability, patient reported outcome measures (PROMs), and return to sports (RTS) rates or activity scores

Stability, patient reported outcome measures (PROMs) are shown in Table 6. Among 16 studies which reported different stability measures, 13 studies used instrumented laxity testing (KT 1000).

**Table 6:** Stability, patient reported outcome measures (PROMs) and return to sports (RTS) rates and activity

Nr	First author, year	Stability	PROMs	Activity, RTS rate	Mean follow up (years)
1.	Shelbourne, 2004	•Lachman: All knees negative •Mean KT-1000 difference: 2.1 ± 1.2 mm (range, 0–4 mm)	•Noyes score: 97.6 ± 2.9 (range, 91–100) IKDC subjective score: 95.4 ± 6.9	•All patients were able to return to high school competitive sports	•3.4
2.	McIntosh, 2006	•Lachman test: 15 patients -, one patient 1 + •Pivot shift: 15 patients -, one patient 1 +	•Lysholm score: 90 (range: 74 to 94) •IKDC subjective score: 99 (range, 94 to 100)	•10 patients (63%) returned to their identical preoperative sport •14 patients (87.5%) returned to their previous level of activity •Tegner activity scale: 8.1 (range, 7 to 9)	•3.4(2–9.3)
3.	Liddle, 2008	•Lachman test: negative in 16 patients and 1 + in 1 patient •Pivot shift: negative in 16 patients and 1 + in 1 patient	•Lysholm score: 97.5 (SD 2.6)	•Tegner activity scale: 8.1 (SD 0.8)	•4.4
4.	Cohen, 2009	•KT-1000 difference: 2.0 + -1.0 mm	•IKDC score: 91.5 ± 5.7 •Lysholm Scale: 93.5 ± 4	•NR	•4.5
5.	Streich et al. 2010	•KT-1000 difference conservative: 4.3 ± 2.9 (SD) mm (range, 2–9 mm), side-to-side difference in KT-1000 difference operative: 1.8 ± 1.4 (SD) mm (mean, range, -1–5 mm)	•Operative group: IKDC median 95, Lysholm 93, conservative group, median of 87 points for IKDC and 84 points for the Lysholm	•Patients with ACL reconstruction: preoperative Tegner 7 ± 1.1, postoperative 7 ± 0.9 ( •Non operative treatment: preoperative Tegner 7 ± 0.7, postoperative 6 ± 0.84	•5.8
6.	Mauch, 2011	NR	NR	NR	5
7.	Nikolaou, 2011	•KT-1000/2000 difference 2 mm (range 0–3.5 mm) •Lachman test: 91patients – or + , 3 patients + + •Pivot-shift test: 2 patients positive (+ +)	Lysholm score: 89 (range 77–100)	•Tegner: 7 (range 5–9) before injury, 3 (range 0–4) pre-surgery 6 (range 4–8) postoperatively •94 patients, 73 (78%) returned to their identical preoperative sport	•3.2(2–5)
8.	Courvoisier, 2011	•KT 1000 difference: 1 mm (SD 1.5)	NR	NR	2
9.	Hui, 2012	•KT-1000 difference: 1.9 mm •Lachman: – 11 patients (69%) had a negative Lachman test finding, + 4 patients •Pivot shift: 14 -, 1 Pivot glide	•Lysholm knee score was 97 (range, 90–100) •IKDC subjective score was 96 (range, 84–100)	•All patients had returned to strenuous activities	•2.1 (1.8–2.8)
10.	Redler, 2012	•KT 1000 difference: 0.29 ± 1.07 mm	•Mean IKDC subjective 92.4 ± 10.0, •Lysholm 94.4 ± 8.8	•Tegner: 8.6 ± 1.4	•3.6 (2–7.2)
11.	Memeo 2012	•Lachman – in all patients •Pivot shift – in all patients	•OAK Score: 87.7	•All patients have returned to their preinjury level of daily activity and athletic participation	•15
12.	Kumar, 2013	•KT 1000 difference: 30 patients < 3mmin, 2 patients 4 to 5 mm	•Lysholm preoperatively 71.5, 86 postoperatively 95	•Tegner preoperatively 4.03, postoperatively 7.66	•6 (4–10.8)
13.	Kohl, 2014	•KT-1000: 5.9 ± 1.2 mm	•Lysholm: 94.0 (range 68–100)	•NR	•4 (2–8)
14.	Calvo et al. (2015)	•KT 1000 difference: 2.58 mm (range, –2.7 to 7 mm)	•Lysholm: 89, IKDC subjective: 86 [*P* = .626	•All patients returned to their previous sports activity •24 patients returned to a level equal or above preinjury level, 3 patients returned to a lower level	•10.6 (10–13)
15	Fauno, 2016	•KT-1000 difference 5.2 mm preoperatively, 1.6 mm at postoperatively	•KOOS subscore symptoms preop. 69.1 ± 14.6, postop. 76.8 ± 15.1, pain preop. 67.2 ± 19.3 postop. 85.7 ± 14.4, activities of daily living preop. 73.8 ± 23.0 postop. 91.0 ± 11.7, sports/recreation preop 8.7 ± 29.1 postop. 69.2 ± 26.8, quality of life 35.4 ± 19.6 67.9 ± 23.9	•Tegner preop. 2.8, postop. 6.1 (	•5.6. ± 2.8
16.	Placella, 2016	•KT-1000 difference preop.5.2 (range: 3.5 to 7) mm, postop.0.8 (range: 0 to 2.5) mm	•Lysholm; 100, IKDC score 98.58 (range: 95.4 to 100	•Tegner: 9.2 before injury, 8.7 after surgery •All patients returned to playing sports after a mean period of •6.43 months (range: 4 to 7 months)	•8
17.	Pennock, 2019	•NR	•Patient satisfaction 9.3 ± 1.1, SANE 91 ± 11, and Lysholm 95 ± 7,	•Tegner: 6.9 ± 1.9	•2.8
18.	Astur, 2019	•NR	•Lysholm score without ACL rerupture 90.6 ± 6.1 v •Lysholm score with ACL rerupture 58.8 ± 6.7	•Tegner without ACL rerupture 7.0 ± 0.8 •Tegner witht ACL rerupture 4.9 ± 1.3, •Patients without ACL reruptures: 46.2% increased their activity, •46.2% returned to their preinjury level of activity, and •7.6% returned to a lower level of activity	•7.4
19.	Gosh, 2020	•KT1000 difference (range) 2.0 (− 1 to 5)	•Median IKDC score/100 (range) 96 (82–100)	•NR	•2
20.	Hanssen, 2022	•NR	•KOOS Symptoms 5 y 86 86 (68–93), Pain 5 y 107 94(83–100), AdL 5 y 107 99 (94–100), QoL 5 y 107 75 (50–88)	•NR	•6.9(5–9)
21.	Kilkenny, 2022	•NR	•IKDC Score 90.3 ± 7.9, 89.3 ± 7.6	•Marx Score 11.9 ± 4.6 11.3 ± 4.6 •Total rate of RTP HT 100% BTB 92.6%	•2

Nineteen studies assessed clinical scores as outcome measures (PROM) such as Lysholm (13 studies), subjective International Knee Documentation Committee (IKDC) score (9 studies), knee osteoarthritis outcome score (KOOS) (two studies), Orthopädischer Arbeitskreis Knie (OAK) score (one study), or the Noyes score (one study).

Nine studies presented return to sport (RTS) rates and 10 studies reported activity as measured with the Tegner activity scale.

## Discussion

The results of this systematic review support our initial hypothesis. There is no difference in the reported failure rates of transtibial (10.8%) and independent drilling (11.4%) techniques. These results underline recent systematic reviews comparing transtibial and independent femoral drilling in ACL reconstruction in adult patients who also found no significant difference in re-injury rate between the two techniques [14, 38]. However, these systematic reviews showed that independent femoral drilling led to reduced instrumentally measurable anterior tibial translation, a lower rate of positive postoperative pivot-shift test findings, and higher patient reported outcome measures (PROMs) such as IKDC subjective score or Lysholm score [14, 38]. Another systematic review could reveal that the rate of post-traumatic osteoarthritis is significantly lower after anatomic ACL reconstruction compared to non-anatomical techniques [12]. Therefore, anatomical techniques for ACL reconstruction are highly recommended in a recently published consensus paper [13]. The independent drilling technique is considered a key feature of anatomical ACL reconstruction, since the femoral insertion zone of the ACL can be better reached via the medial portal than via the tibial tunnel [9, 10]. The most commonly used independent drilling technique is the medial portal drilling technique [14].

In children with open growth plates, independent drilling techniques were initially viewed critically because the more horizontal course of the femoral tunnel might cause more damage to the growth plate [5, 39]. An MRI study has shown that femoral tunnels created with an independent tunnel drilling technique disrupt a larger area of the distal femoral physis and create more eccentric tunnels compared to a transtibial technique. Interestingly, in the present systematic review, no increased rates of growth disturbances such as leg length discrepancies or angular deformities could be found in studies on independent drilling technique. Growth disorders requiring treatment and those not needing treatment were detected more frequently in the transtibial group.

The study by Fauno et al. has shown, that disturbances of the growth plate can also occur after transphyseal ACL reconstruction without observable leg length discrepancies or angular deformities [20]. In contrast to many other scientists, Fauno et al. not only considered the leg length or the mechanical femorotibial leg axis, but also joint angles, namely the medial proximal tibial angle and the lateral distal femoral angle [20]. In their study a slight valgus deformity was observed on the operated side but the difference was not statistically significant [20]. However, it was found that compared to the contralateral side, the distal femoral joint angle showed significantly more valgus, whereas the medial proximal tibial angle displayed more varus [20]. The authors interpreted these results to mean that the measurable femoral valgus deformity was counterbalanced by the tibial varus deformity, leading to a non-significantly increased number of patients with valgus deformity in the femorotibial leg axis [20]. The long-term consequences of the resulting joint line obliquity are unknown. The rate of leg length discrepancies > 1 cm reported in Fauno's study is also a cause for concern. [20] Therefore, this study shows that transphyseal drilling is not as benign as primarily thought. Randomized controlled studies are needed to evaluate growth disturbances after transphyseal drilling in more detail. The present systematic review would warrant a randomized controlled trial comparing transtibial and independent drilling techniques.

Another concern are the overall failure rate of 11.0% and the rate of contralateral ACL tears. This re-injury rate is higher than re-injury rates found in previous systematic reviews [40, 41]. This may be due to the fact that previous systematic reviews also included other techniques than transphyseal ACL reconstruction and that an increasing number of studies have been published in recent years that have reported mid-term and long-term results [41, 42].

A new surgical strategy to reduce the failure rate in pediatric ACL reconstruction utilizes lateral extraarticular tendodesis (LET). Recent studies have shown that adding a LET to an all epiphyseal or transphyseal ACL reconstruction can reduce the re-injury rate significantly [43, 44]. A recent randomized study has shown that in young patients (not pediatric) at high risk of failure, the addition of an anterolateral tenodesis to a single-bundle ACL reconstruction resulted in a statistically significant, clinically relevant, reduction of graft ruptures and rotatory laxity [45].

In recent years there has been an increasing interest in lateral extra-articular procedures in pediatric patients with an ACL injury [46, 47]. A recent survey among American orthopaedic surgeons has shown that 56% performed an anterolateral augmentation with primary ACL reconstruction in rare cases, and 79% in revision ACLR. More studies are needed to evaluate the role of lateral extraarticular procedures in pediatric patients.

Regarding the reduced number of failures, the comparison of failure rates of the various autologous tendon grafts is relevant, too. Interestingly, failure rates were significantly lower when using grafts from the extensor apparatus (PT or QT) than when using a HT. This might have various causes. The diameters of flexor tendons in children are often pretty small and it is well known that a small transplant diameter is associated with a significantly higher risk of graft failure [48]. Another reason may be the preservation of the flexor tendons, which are known to act agonistically to the ACL and limit anterior tibial translation [49]. However, both factors can not explain that even the group with HT grafts from living donors shows a higher rerupture rate than the extensor tendon group (diameter of an adult tendon, not harming the agonistic flexor tendons). It might be an explanation that the inhibition of the extensor mechanism plays an important role, since it also plays an important role in the occurrence of an ACL injury. Since groups of patients who received a QT or BTB graft is small, more research is needed to evaluate the potential of both graft sources for pediatric ACL reconstruction. There is special interest in the QT, as it can be harvested in sufficient length without a bone block [50]. It is not recommended to use bone blocks for pediatric ACL reconstructions, as they can bridge the growth plates and therefore lead to growth disturbances [5]. For example, the femoral valgus deformity described by Mauch et al. requiring an osteotomy was caused by a bone block bridging the growth plate [28]. The results of this systematic review indicate that autologous QT is a promising graft option for pediatric ACL reconstruction.

Also of concern is the high rate of contralateral ACL ruptures. The same observation was made after ACL reconstructions in adults [51]. It should be noted that all patients with an ACL rupture are generally considered as risk patients [52]. Since certain risk movement patterns can be corrected by special prevention programs, it should become daily routine to screen all ACL patients for these risk movement patterns [52].

As for many systematic reviews, some limitations apply for the present study. One limitation is that case series only could be included in the review and that evidence, gained from these studies, is generally rated as low. But even if the level of evidence of the included studies is low, case series should not generally be excluded from finding scientific evidence [53]. The inclusion of case series in systematic reviews seems to be justified when no other higher level of evidence such as randomized controlled trials is available [54]. Nevertheless, randomized controlled studies about pediatric ACL reconstruction are needed. Another limitation of this study could be that the bone age or Tanner stage of the patients was not specified in all studies. However, since the upper age limit of 16 years was chosen as an exclusion criterion, we believe that mainly patients with open growth plates were included.

## Conclusion

Despite the mentioned limitations this systematic review shows that children and adolescents have a relatively high failure rate after transpyseal ACL without any statistically significant differences between independent or transtibial drilling techniques regarding reruptur rates or the rate of growth disturbances. The results of this systematic review warrant a comparison of both techniques for femoral tunnel drilling in a controlled randomized trial a controlled randomized trial.

## Data Availability

Data obtained during the literature review by the independent authors are available upon request.
